# Spirituality: A Key Component of the Salvation Army’s Bridge Programme Model of Treatment in Aotearoa New Zealand

**DOI:** 10.1007/s10943-022-01674-7

**Published:** 2022-09-29

**Authors:** Richard Egan, Julien Gross, Claire Cameron, Linda Hobbs, Tess Patterson

**Affiliations:** 1 Department of Preventive and Social Medicine, University of Otago, PO Box 56, Dunedin, 9054 New Zealand; 2 Department of Psychology, University of Otago, Dunedin, New Zealand; 3 Biostatistics Centre, University of Otago, Dunedin, New Zealand; 4 Department of Psychological Medicine, University of Otago, Dunedin, New Zealand; 5 Opentia Research Focus Area, North-West University, Potchefstroom, South Africa

**Keywords:** Substance use disorder, Alcohol and other drug, Spirituality, The Salvation Army Bridge Programme, Recovery, Religion, Addiction, New Zealand

## Abstract

Spirituality is vital to The Salvation Army’s Bridge model of treatment for alcohol and drug addiction. Spirituality is expressed through Recovery Church, prayer, spirituality lifters, the 12-step programme, and focuses on meaning and purpose. We recruited participants from several regional centers throughout Aotearoa New Zealand and evaluated spirituality using the WHOQol-SRPB and open-ended questions. Most participants held broad understandings of spirituality, only a minority equating it with religion. Participants who completed the Programme had statistically significant increases in spiritual wellbeing at end-of-treatment. These increases were maintained at a 3-month follow-up. Increases in spiritual wellbeing were associated with decreases in severity of alcohol and drug use.

## Introduction

There is increasing interest in the role that spirituality may play in the addiction recovery process, with evidence suggesting that a strong sense of spirituality may offer mental and physical health benefits for those in recovery (Amaro et al., 2010; Avants & Margolin, 2004; Baker et al., 2007; Culliford, 2011; Kelly & Eddie, 2020). High personal levels of spirituality or religiosity may act as a protective factor against substance use and abuse for both adolescents and adults (Hodge et al., 2001; Larson & Larson, 2003; Miller, 1999; Ritt-Olson et al., 2004; Temme & Kopak, 2016), while within treatment settings, spiritual or religious support may improve the success of substance abuse programmes (Avants et al., 2001; Flynn et al., 2003; Polcin & Zemore, 2004; Zemore & Kaskutas, 2004). For example, spiritual or religious support for patients undergoing methadone treatment was predictive of abstinence from heroin and cocaine (Avants et al., 2001), with abstinence maintained for up to five years post-treatment (Flynn et al., 2003). Similarly, a strong personal sense of spirituality has been found to be predictive of sobriety among recovering alcoholics (Polcin & Zemore, 2004; Zemore & Kaskutas, 2004). This growing body of evidence has led some researchers to suggest that “it is becoming increasingly difficult to exclude spirituality as a possible factor in the addiction recovery process for many individuals” (Stewart, 2008, p. 402). The International Society of Addiction Medicine suggests spirituality is part of one’s “recovery capital” and recommend assessment of spirituality (Galanter et al., 2021).

However, there is evidence that patients and clinicians differ in the importance they place on spirituality as part of the recovery process. For example, nurses were reported to undervalue the importance of spirituality during the recovery process for residential patients (McDowell et al., 1996), instead prioritizing food, accommodation, and entertainment as factors crucial to the recovery process. Similarly, both medical students and medical teaching staff were found to underestimate the importance of spirituality for patients, instead focusing on the importance of employment opportunities and outpatient treatment programmes (Galanter et al., 2007). In contrast, many of those in recovery, or those working in the addictions area, have highlighted the importance of spirituality in rehabilitation (Treloar et al., 2014). There may also be differences in the ways that patients and many clinicians conceptualize successful outcomes or recovery. Some spirituality-based approaches to addiction treatment frame recovery according to the personal experiences of the substance users (Galanter, 2007). “Recovery” is viewed and defined “through the prism of the person’s own introspection and reflection” (Galanter, 2007, p. 266), as opposed to biomedical and behavioral psychology approaches which focus on the attainment and maintenance of sobriety. Moreover, in “spiritual grounded recovery,” the goal of treatment is “the achievement of meaningful or positive experiences, rather than a focus on observable, dysfunctional behaviors” (Galanter, 2007, p. 266). Other researchers have cautioned that spiritual needs are highly individualized and that clinicians should not assume that the 12-step programme or other spirituality-based treatment will appeal to all patients (Arnold et al., 2002; Dermatis et al., 2004; Neff et al., 2006). Nevertheless, many researchers and practitioners suggest that spiritual or religious components, such as private prayer, meditation, spiritual readings, and discussions of meaning and purpose in life should be included in treatment programmes (Kelly & Eddie, 2020; Koenig et al., 2001; Piderman et al., 2008; Wills et al., 2003).

The most widespread spirituality or faith-based addiction programme is Alcoholics Anonymous (AA) and its sister programmes (e.g., Narcotics Anonymous), a non-denominational approach based on the incompatibility of substance use and spirituality (Miller, 2002). AA is based on fellowship and the sharing of experiences among members, with the 12-steps that form the basis of the programme focused on facilitating spiritual growth. AA most commonly operates through a worldwide network of community-based fellowship meetings but is also often one of a range of treatments offered to patients in formal recovery programmes (Stewart, 2008). Attendance at AA meetings has been associated with increased levels of spirituality or religiousness, which may also impact on substance use levels (Kelly et al., 2011). A theory that has been advanced to explain AA’s success is that the programme may reduce “oppositional barriers” to spiritual exploration (Kelly et al., 2011), which in turn increases overall psychological wellbeing that subsequently encourages and reinforces attitudinal and behavioral changes beneficial for recovery (Kelly et al., 2010; Pearce et al., 2008).

However, there is still considerable debate regarding the efficacy of AA or why the programme is efficacious (Segal, 2020). For example, an AA membership survey conducted in 2014 found that 73% of members who responded to the survey reported having been sober for at least one year (Alcoholics Anonymous, 2014), other surveys however have reported much lower rates of success (Tonigan et al., 2002). Furthermore, as the programme includes additional provision of ongoing, community-based support, it is seen to provide essential “boosters,” which some researchers have suggested may help reinforce and sustain behavioral and attitudinal changes beyond that of just spiritual exploration that are necessary to maintain sobriety (Richard et al., 2000; Tonigan et al., 2002). Additionally, others argue that AA facilitates adaptive social networks which is what leads to enhanced good outcomes (see Segal, 2020, for full discussion). Although overall, AA is viewed as efficacious, it is also not without criticisms. In particular, the AA programme is theistically-based: several of the steps explicitly mention “God *as we understand Him*” (emphasis in the original) (Segal, 2020), which may limit the appeal of the programme for some individuals (Brown et al., 2006; Galanter, 2007). Researchers have also identified a desire among some patients for spirituality-based therapies that extend beyond the 12-step approach and beyond a theistic God (Dermatis et al., 2004).

The Salvation Army, a Christian organization operating across many countries, incorporates the 12-step approach in the provision of residential and community-based alcohol and other drug (AOD) rehabilitation treatment. A review of The Salvation Army’s AOD programmes in the USA reported that participation in the spirituality components of the programme may be associated with treatment completion (Wolf-Branigin & Duke, 2007), while an Australian study reported that 75% of the clients at a local Salvation Army treatment center found the spiritual and religious aspects of the programme useful in their recovery (Mason et al., 2009).

In Aotearoa New Zealand, The Salvation Army offers the Bridge Programme Model of Treatment (“Bridge Programme”); an 8-week multicomponent intensive treatment service for adults with alcohol and other drug addictions. The Salvation Army operates the Bridge Programme in 14 AOD treatment centers nationwide, providing support to approximately 1500 clients each year. The programme comprises four key components: Partnership, the Community Reinforcement Approach, the Twelve Step Facilitation Programme, and The Salvation Army spiritual elements. For a summary of Aotearoa New Zealand’s spiritual and religious context, see Baker et al. (2007) and for an overview of Māori wairua/spirituality, see Valentine et al., (2017). Several elements of the Bridge Programme are explicitly spiritual or religious in nature. These include Recovery Church, use of prayer and guidance, spirituality classes, and the Twelve Step Facilitation Programme (a treatment approach based on the premise that an alcohol or drug problem is a disease that can be addressed across 12 key steps involving meetings with peers, and recognition of a higher power to support the change process, Nowinski et al., 1992). The present study was part of a larger nationwide study evaluating the effectiveness of the Bridge Programme provided by The Salvation Army New Zealand, Fiji, Tonga and Samoa Territory (see Patterson et al., 2015, for full details). In the present study, we explored the role that spirituality and religion may play in the addiction recovery process for clients of the programme.

## Method

### Participants

Recruitment for the study took place at seven Salvation Army centers nationwide over a 12-month period beginning in January 2014. Participating centers were chosen by the National Manager of The Salvation Army’s Addiction and Supportive Accommodation Services and the National Operations Manager of The Salvation Army’s Bridge Programme. The centers were chosen because they both delivered the Bridge Programme and were representative of the range of clients that utilize the Bridge Programme, including centers located in larger and smaller urban areas, and centers’ with rural clients within their catchment area. During the 12-month period, 675 clients seeking drug and alcohol recovery treatment were invited to participate in the evaluation study. Participants were eligible to participate in the study if they were over the age of 18, entered the programme at one of the participating centers during the recruitment period, had not participated in the Bridge Programme within the previous three months, and gave written informed consent to participate. Ethical approval for the procedures used in the evaluation was received from a New Zealand University’s Human Ethics Committee and from the local Regional Ethics Committee, both of which are approved by the New Zealand Health Research Council and whose guidelines are consistent with those of the American Psychological Association.

A total of 325 participants *(*mean age = 39.60 years; range 20 years to 73 years; 64% male) provided written informed consent to participate. The majority of the participants identified themselves as NZ European (66%) or Māori (31%), 10% identified Pacific Island ancestry (Samoan, 4%; Cook Island Maori, 3%; Tongan 1.5%; and Niuean, 1%), the remainder identified as Indian (1.5%), Chinese (< 1%), or another (7%) ethnicity.^1^ At the end-of-treatment, participants were asked if they belonged to a church, synagogue, or mosque. A total of 52 (23% of those who completed treatment) participants answered yes (including 10 who said they belonged to The Salvation Army’s Recovery Church throughout treatment). Of the 52 who said that they belonged to a church, synagogue, or mosque, 31 said that they attended weekly or monthly. The remaining said they attended either every few months, seldom, or never.

### The Salvation Army Bridge Programme

As per the standard treatment protocol for the Bridge Programme, all clients underwent a comprehensive assessment at entry to the programme. Those clients who gave written consent to participate in the study also completed a full set of outcome measures at three specific time points: (1) baseline (before treatment), (2) at the end-of-treatment, and (3) at follow-up, 3-months after graduation from the programme or leaving the programme. The measures were completed at a Bridge Programme center. A research assistant supervised each session and if required, provided support with reading and writing.

### Outcome Measures

We used a battery of psychometrically-validated measures to evaluate primary and secondary outcomes (see Patterson et al., 2015, 2018 for full details). For the purpose of this study, we report only the results from four quantitative and qualitative measures: (1) spiritual wellbeing (the WHO Quality of Life-Spirituality, Religiousness and Personal Beliefs (WHOQoL-SRPB; WHOQoL SRPB Group, 2006)); (2) Addiction Severity Inventory (ASI; McLellan et al., 1992) domain scores for severity of ‘alcohol use’ and ‘drug use’; and participants’ answers to (3) a checklist of definitions of spirituality, and (4) a series of open-ended questions about spirituality.

In total, 225 participants with a mean age of 40.69 years (standard deviation = 11.57; range 20 years to 73 years; 65.2% male) completed sufficient treatment to be considered as having received a therapeutic dose. A “therapeutic dose” of treatment was considered to be more than 28 consecutive days of attendance or at least half or more of the treatment programme. Specifically, 182 participants completed treatment and graduated from the programme, and 43 participants did not graduate but completed a substantial portion (more than 28 days) of the 8-week programme. Of those who completed a dose of treatment, 171 (76%) completed the outcome measures at the end-of-treatment, and 108 (48%) completed outcome measures at the 3-month follow-up.

#### Spiritual Wellbeing

We used the WHO Quality of Life-Spirituality, Religiousness and Personal Beliefs (WHOQoL-SRPB; WHOQoL SRPB Group, 2006) instrument to assess the spiritual aspects of the participants’ quality of life at baseline, end-of-treatment, and at the 3-month follow-up. The WHOQoL-SRPB is a 32-item self-report measure designed to evaluate how spirituality, religiosity, and personal beliefs (SRPB) are related to quality of life in health and health care (Fleck & Skevington, 2007) within the previous two weeks. The WHOQoL-SRPB is applicable to people from a variety of cultures, and spiritual and religious traditions. Individuals rate their agreement with each item on a 5-point scale (1 = *Not at all* to 5 = *An extreme amount/Extremely/Completely/Very satisfied*). The items are grouped into eight facets (spiritual connection, meaning and purpose in life, experiences of awe and wonder, wholeness and integration, spiritual strength, inner peace, hope and optimism, faith). Responses are summed and mean facet-specific scores and an overall mean WHOQoL-SRPB score are calculated with higher scores indicating higher perceived quality of life within the previous two weeks. Scale reliability analyses on participants’ baseline scores revealed that all 32 items formed a reliable scale (Cronbach’s *α* = 0.97, *M*_inter-item correlation_ = 0.49); Cronbach’s *αs* for the facet-specific scores ranged from 0.77 (wholeness and integration) to 0.96 (faith).

#### Severity of Use

We measured the severity of participants’ substance use problems using a standardized computer-administered assessment, the Addiction Severity Index–Multimedia Version (ASI-MV^®^; Inflexxion, Irvine, CA, USA). The ASI-MV is based on the interview version of the Addiction Severity Index (ASI), an assessment tool widely used in the evaluation of problem severity in individuals in substance use treatment (McLellan et al., 1992). The ASI-MV measures severity ratings in seven biopsychosocial domains associated with substance use: medical, employment, legal, family, psychiatric, alcohol, and drug use. Here, we only considered composite (recent) scores calculated for the ‘alcohol’ and ‘drug use’ domains. Higher scores indicate greater need for treatment. The ASI-MV has demonstrated reliability and validity (Butler et al., 2001).

#### Subjective Views of Spirituality

At the end-of-treatment, participants were asked to complete a written questionnaire relating to spirituality. First, participants were asked to provide their own open-ended definition of what spirituality meant to them by answering the question, “How would you define spirituality?” Next, participants were asked to define what spirituality meant to them by selecting as many responses as applicable from a list of 19 pre-defined conceptualizations of spirituality: faith, beliefs, values, meaning, purpose, sense of awareness, balance, God, identity, inner core, life giving force, relationships, connectedness, essence, mystery, religion, transcendent, it is meaningless, and other. Finally, participants were asked to provide written responses to the following six open-ended questions: (1) Do you have a belief system that is important to you? Has this changed since finishing the Programme? (2) What matters to you most? Has this changed since finishing the Programme? (3) Did the Programme affect your spirituality or religious beliefs? If so, how? (4) Did the Programme alter any sense of meaning or purpose in your life? If so, how? (5) Are there spiritual practices (e.g., prayer, attendance at church) that have helped you during your time in the Programme? If so, what? (6) How did the discussion about knowledge of a higher power affect your experience in the Programme?

### Analysis

All analyses were conducted on participants who received a ‘dose’ of treatment (*n* = 225). Measures are reported at three time points (baseline, end-of-treatment, and 3-month follow-up).

#### Spiritual Wellbeing and Severity of Use

We calculated an overall mean WHOQoL-SRPB score and eight facet-specific scores for each participant at each of the three time points. The ASI-MV provides calculated composite (recent) scores for seven separate domains but we only used the ‘alcohol use’ and ‘drug use scores.’ We used linear mixed models (adjusting for participant sex, age, time in treatment, and reason for referral) to assess the effect of treatment on spiritual wellbeing, and the relation between spiritual wellbeing and our primary outcome measure, severity of use. All models had a random effect on (a) treatment center (city), to allow for clustered observations, and (b) individual, to allow for repeated observations. The models allowed us to determine whether outcome changed from baseline to end-of-treatment or 3-month follow-up. Adjusted means and 95% confidence intervals are presented.

The models evaluating severity of alcohol and drug use and its relation to the spirituality overall score were similar in structure to the ones just described. However, the outcome was the alcohol or drug use severity and the overall spirituality score was a predictor. In each case, the model was restricted to those who were referred for alcohol use or drug use, respectively. We adjusted for age, sex time in treatment and phase of treatment and we included a random effect for treatment center and individual.

#### Subjective Views of Spirituality

We collated and counted the frequency of ideas contained in participants’ responses to the question, “How would you define spirituality?” We also calculated the number (and percentage) of participants who selected each of the 19 pre-defined conceptualizations of spirituality. As participants were able to select more than one definition, percentages exceed 100%.

The first author collated and thematically coded (Caelli et al., 2003) the responses to the six open-ended questions on the spirituality questionnaire. The codes were based on the questions (e.g., meaning and purpose) and the researcher’s prior knowledge (e.g., values). Further analysis was done in partnership with other researchers on the team (TP, JG), therefore some comparison and discussion of findings has added to these results. We analyzed Questions 1 to 4 to examine the Bridge Programme’s impact on the participants and their sense of spirituality, Question 5 to examine specific spiritual practices that may have been helpful to participants and finally, Question 6 to examine higher power discussions. We illustrate our results using direct quotes from participants. Individual participants are indicated by the numbers in parentheses following the quotes.

## Results

### Spiritual Wellbeing Across Baseline, End-of-Treatment and 3-Month Follow-Up

Table 1 shows that the overall WHOQoL-SRPB adjusted mean scores increased from baseline (mean = 10.20 (95% CI: 9.14, 11.25) to end-of-treatment (12.50 (11.42, 13.59)), indicating that participants’ spiritual wellbeing increased over the course of the treatment programme. At the 3-month follow-up, WHOQoL-SRPB adjusted mean scores had decreased slightly (11.90 (10.77, 13.07)) in comparison with end-of-treatment, indicating a reduction in spiritual wellbeing. In this model, the change in mean score from baseline to end-of-treatment and from baseline to the 3-month follow-up were important (*p* < 0.01). There was a significant reduction (*p* = 0.045) in spiritual wellbeing at the 3-month follow-up compared to at end-of-treatment.

**Table 1:** WHOQoL-SRPB Overall and Facet-Adjusted Means for Baseline, End-Of-Treatment, and Three-Month Follow-Up

Facet	Baseline mean (95% CI)	End-of-treatment mean (95% CI)	3-month follow-up mean (95% CI)	*p-value* comparing 3-month follow-up to end-of-treatment
Spiritual connection	2.09 (1.73, 2.46)	2.65 (2.27, 3.02)	2.43 (2.04, 2.82)	0.032
Meaning and purpose	3.23 (2.96, 3.51)	3.59 (3.31, 3.87)	3.51 (3.21, 3.81)	0.382
Experience of awe and wonder	2.87 (2.62, 3.13)	3.24 (2.97, 3.50)	3.22 (2.94, 3.49)	0.797
Wholeness and integration	2.52 (2.26, 2.79)	3.20 (2.94, 3.47)	3.05 (2.77, 3.34)	0.083
Spiritual strength	2.23 (1.88, 2.58)	2.85 (2.49, 3.20)	2.63 (2.26, 3.00)	0.032
Inner peace	2.27 (1.97, 2.58)	3.11 (2.79, 3.43)	2.90 (2.57, 3.24)	0.042
Hope and optimism	3.05 (2.80, 3.31)	3.58 (3.32, 3.85)	3.47 (3.19, 3.74)	0.169
Faith	2.13 (1.75, 2.52)	2.79 (2.40, 3.18)	2.58 (2.17, 2.98)	0.039
Overall: WHOQoL-SRPB	10.20 (9.14, 11.25)	12.50 (11.42, 13.59)	11.90 (10.77, 13.02)	0.045

CI = 95% confidence interval. Estimates are adjusted for gender, age, use classification, and treatment length (either completed the treatment or did not graduate, but attended more than 28 days of stage 2)

Table 1 shows that adjusted mean scores for each of the eight WHOQoL-SRPB facets also changed over time. Statistical modeling established that participants’ scores for all eight facets of spiritual wellbeing increased significantly (an increase in score indicated an increase in spiritual wellbeing) from baseline to end-of-treatment (*p* < 0.01), and from baseline to the 3-month follow-up (*p* < 0.01). Between end-of-treatment and the three-month follow-up, however, there were significant reductions in scores on four of the facets, spiritual connections, spiritual strength, inner peace, and faith (shown in Table 1).

### Spiritual Wellbeing and Severity of Alcohol and/or Drug Use

Statistical modeling showed that for participants who were referred for alcohol use (i.e., alcohol only, or alcohol and drugs), a decrease in severity of alcohol use (ASI-MV composite ‘recent’, domain: ‘alcohol use’) was associated with an increase in overall mean score on the WHO QoL-SRPB. The adjusted mean *decrease* in alcohol severity was 0.011 (95% CI: 0.004, 0.018) for a one unit increase in the spirituality scale. Likewise, for participants who were referred for drug use (i.e., drugs only, or drugs and alcohol), a decrease in severity of drug use (ASI-MV composite ‘recent’, domain: ‘drug use’) was associated with an increase in overall mean score on the WHOQoL-SRPB. The adjusted mean *decrease* in drug severity was 0.005 (95% CI: 0.002, 0.009).

### Participants’ Definitions of Spirituality

Participants were asked to provide their own definition of spirituality, and these answers included energy, justice, wairua (an important concept for many people of Māori ethnicity, the indigenous people of Aotearoa New Zealand, which is commonly translated as “spirit of a person,”; Nelson-Becker & Moeke-Maxwell, 2020), freedom, self-love, higher power, love, tolerance, non-judgmental, and acceptance.

**Fig. 1:**
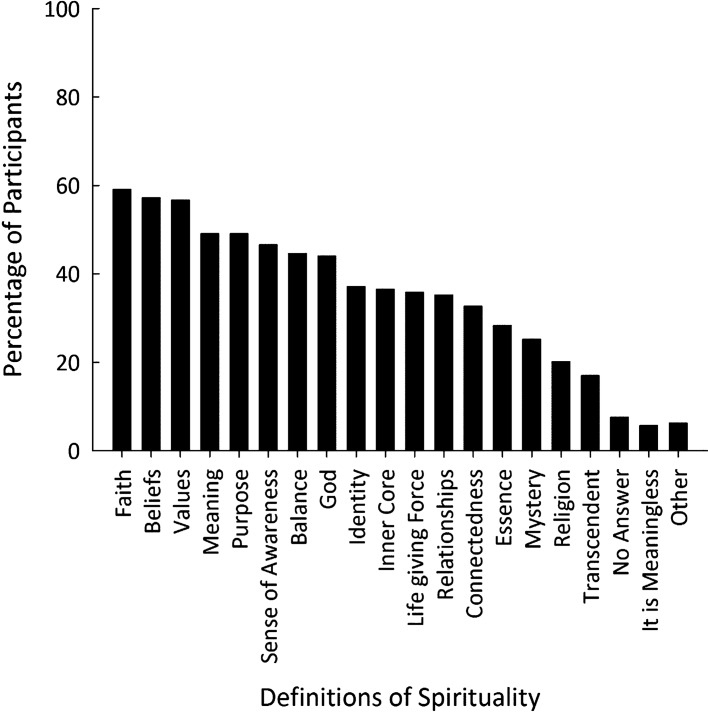
The Percentage of Participants Who Selected Each Definition of Spirituality. *Note* Participants could select more than one definition of spirituality.

Participants were also asked to define what spirituality meant to them by selecting from a list of pre-defined conceptualizations of spirituality. Figure 1 shows the percentage of participants who selected each of the 19 pre-defined conceptualizations of spirituality presented to the participants at end-of-treatment. Overall, participants selected an average of 6.86 (*SD* = 5.32) definitions with a median of 6. The most frequently chosen definitions were faith (59%), beliefs (57%), values (56%), meaning (49%), and purpose (49%). Only 5% of participants selected “it [spirituality] is meaningless.”

### The Bridge Programme and Its Impact on Participants and Their Sense of Spirituality

At the broadest level, the thematic analysis of the open-ended questions 1 to 4 fell into two main types of responses: those that were explicitly religious and those that were more “generically” spiritual (i.e., not phrased in religious language). Further analysis revealed the themes of discovery, affirmation, ambivalence and rejection, with a number of sub-themes emerging. These included the importance of sobriety, beliefs and values, awareness, connection, meaning and purpose in life, contribution, compassion, optimism, and acceptance.

### Discovery

#### The Importance of Sobriety

While attaining and maintaining sobriety is a key outcome of the Bridge Programme, for several participants maintaining their sobriety was the most important thing in their lives:

My sobriety is the most important thing to me. I have different priorities now. I was all about me when I was drinking. _(1004)_

My sobriety. It has started since finishing the programme. This encompasses spirituality, relationships, self-awareness, pride. _(812)_

#### New Beliefs and Value Systems

A number of participants wrote about discovering new beliefs and value systems after participating in the Bridge Programme, with some participants expressing explicitly religious beliefs and values:

I was a non-believer. Now I believe in God who I choose to be my higher power and will continue to have faith. _(1007)_

God is very important to me. I only found him here. _(1039)_

At first I didn’t want to know but now I have open[ed] my heart to the Lord. _(554)_

For other participants, the Bridge Programme had led to the discovery of new aspects of their faith. For example, one participant with Islamic beliefs wrote that life *“became more clearer”*_(127)_ by the end of the programme, while a church-attending participant said that his beliefs had become *“more expansive”* and that what mattered most in his life now was *“being warm and worthwhile to all … finding God’s Spirit in other people”*_(135)_.

Many participants also wrote about discovering a more general sense of spirituality, or of developing a new understanding of spirituality that was distinct from religion:

It made me aware of spirituality … gave me a sense of belonging and a spiritual awakening. _(113)_

It gave me a better understanding of it (it’s not just God, it can be anything). _(320)_

#### Awareness

Many participants also commented about greater self-awareness:

Yes, it has made my spiritual life stronger by understanding my addiction and myself. _(129)_

Greater awareness of spiritual bankruptcy. I now can say that I have a greater sense of enlightenment. _(723)_

This included the discovery and awareness of a personal Higher Power:

It introduced me to higher power which I tried to get in touch to during the programme. Higher power supported me and help[ed] me a lot. _(537)_

Couldn’t understand at first but it came slowly that it was something outside myself. _(522)_

Increases in awareness also extended beyond the self, and included the consequences of their addiction:

It has taught me to look outside myself. _(1021)_

Being reminded of the devastation the addiction leaves. _(130)_

#### Acceptance

For several participants, the awareness of a Higher Power also helped lead them to acceptance of their addiction and recovery process:

I realized I could rely on my higher power to help me get better. _(301)_

…coming to realize I was powerless over my addiction. _(307)_

#### Connection

The connection to family/whānau was a prominent theme that ran throughout many participants’ responses, often coupled with finding new meaning and purpose in life:

The purpose and meaning in my life is my children. _(555)_

I didn’t really have a purpose until now and that’s be a great dad. _(750)_

Other participants wrote about a more general feeling of belonging:

I have a connectedness with something now where I did not before. _(1027)_

… a sense of belonging and hope that things will work out the way they are meant to be. _(301)_

#### Meaning and Purpose in Life

Participants often wrote about discovering new meaning and purpose in their lives:

Yes, that I have meaning for myself and others around me and what my purpose is in life, by learning that drugs, alcohol is stopping me living fiscally, spiritually. _(129)_

I realize there is a meaning and purpose to my life, before my life felt meaning[less]. _(301)_

#### Contribution

For some participants, the opportunity to reflect on themselves and others led to a feeling of wanting to make a contribution to society:

I feel connected … I want to help other addicts. _(313)_

Yes, helping others, I’ve always wanted to do, I don’t know what area through. But I still want to. _(524)_

#### Compassion

Many participants wrote about discovering a sense of compassion for themselves and others:

Yes, now I believe that I am worthy of a good, happy, full life. I have a sense of direction. _(150)_

Far more compassion is embodied in my system now, both for myself and others. _(1027)_

For two participants, their compassion extended toward forgiveness of God:

I used to blame God for taking my husband and son from me and I resented him for that. Getting rid of this resentment and accepting God has given me peace. _(1013)_

I reconnected again. My resentment towards him has gone. _(1038)_

#### Optimism

Finally, optimism was a thread that ran through many participants’ responses:

It made me feel that I could have a meaningful life without alcohol and drugs. _(119)_

…enjoying life. The world is what you make it. When I started I just thought the world was fucked. _(549)_

### Affirmation

#### Beliefs and Values

A number of participants wrote about how participating in the Bridge Programme had reinforced their existing beliefs and values, whether from an explicitly religious perspective, or from a broader spirituality:

It has resolved my reason for becoming a Christian in the first place. _(130)_

My spirituality has increased hugely. I have always been a spiritual person but I was just tapping on the door. Now the door is open. _(812)_

### Ambivalence

A number of participants expressed ambivalence about the Bridge Programme and whether the programme had affected their sense of spirituality. This included ambivalence about the importance of sobriety itself:

I still believe I need my cannabis for my pain relief … I won’t smoke when I finish my programme. I stay in pain. _(554)_

The Bridge Programme appears to have increased some participants’ awareness of spirituality without impacting on the person’s beliefs. For example, one person admitted they were *“willing to accept the ideas but never took [them] on board”*_(1425)_, while another wrote *“Only in that I pondered it more than I usually would. I have not drawn any conclusions.”*_(753)_

Other participants wrote of how the Bridge Programme had turned them off of religion, but may have opened them to consider the value of spirituality more generally:

It put me off religion a lot, but gave me an awareness of spiritual awareness. _(1028)_

Discussions about Higher Power in the programme put me off it and closed my mind to it. NA [Narcotics Anonymous] and people in NA opened my mind again. _(1050)_

Another participant, while writing about their ambivalence, nevertheless also revealed greater awareness and hinted at optimism for the future:

Just made me think about it more. Made me aware of what’s out there. Still struggling to believe. Time, I have to be patient. _(1004)_

### Rejection

Finally, the spiritual and religious components of the Bridge Programme did not resonate with some participants. One person admitted that it was *“tedious, relentless”*_(120)_, while another wrote about the incompatibility of the programme with their personal beliefs:

I don’t believe in God so it was hard looking at it. _(1407)_

Other participants, many of whom did not complete the Bridge Programme, wrote about feeling confused, with one participant writing *“confusing—didn’t experience it.”*_(1036)_

### Spiritual Practices That May Have Been Helpful to Participants

Question 5 of the questionnaire asked, “Are there spiritual practices (e.g., prayer, attendance at church) that have helped you during your time in the Programme? If so, what?” Participants’ responses include religious responses related to prayer and church; then a whole range of responses related to prayer generically, meditation, wairuatanga (Māori spirituality), and spiritual lifters/classes.

Many of the participants noted the help that they received from Recovery Church and bible readings. One participant suggested that Recovery Church added meaning and purpose.

Prayer was often noted as being helpful and important, as was meditation. Others noted specifically, the “serenity prayer” was particularly helpful. For example, one participant noted, *“I pray in the morning when I wake up and before I go to bed to thank my higher power for my day.”*_(1400)_ Related to this was a sense of surrendering oneself that many felt, *“Surrendering my life and will to my higher power.”*_(1423)_

The programme offered something called a *“spirit lifter”*_(502)_ that happens in the mornings for those living in. This was appreciated by some, as was *“spirituality class.”*_(150)_

While wairuatanga or Māori spirituality practices, such as *“haka”* (ceremonial Māori war dance or challenge) _(157)_ and *“karakia”* (form of prayer) _(509)_ were noted by a small group of participants, generally this was notably absence from participants’ responses. Those who said there were no spiritual practices per se that were helpful made comments such as, *“No, but it was nice to be in a loving atmosphere.”*_(144)_

### Higher Power Discussions

The final question (question 6) asked participants “How did the discussion about knowledge of a higher power affect your experience in the Programme?” Like many of the participants’ responses to questions 1 to 5, answers to question 6 also were either explicitly religious or more generic considerations. Within these groups, the responses were generally positive and focused on letting go/surrendering, a better understanding of a higher power, and a springboard to awareness of spirituality generally.

In the explicitly religious group, some made the direct link between a higher power and God, as these quotes suggest,

God is very important to me. I only found him here. _(1039)_

… it was God that got me here to do this programme. _(108)_

As noted by some of these participants, over the course of the programme, they “found” God or religion, thus it was, in a sense, a conversion experience.

The more generic spiritual responses to the higher power discussion about how this impacted on their programme experience were largely positive,

... key to enjoy life with my higher power. _(122)_

It made me realize there is something bigger than me. _(132)_

… made me aware of my own spiritual perspective. _(301)_

Of note was one participant who suggested that the higher power discussions *“informed me that Higher Power didn’t mean ‘god’”*_(134)_, suggesting the “god of your understanding” (12 Step definition) approach was useful. Other participants found the higher power discussion of no consequence, saying, *“it didn’t”*_(103)_ affect their experience; it was “*tedious, relentless”*_(120)_, it *“Didn’t affect me much but I’m ok seeing it as collective consciousness.”*_(144)_

## Discussion

Using validated quantitative psychometrics of spirituality (i.e., WHOQoL-SRPB and ASI), the present study demonstrated an increase in spiritual beliefs by the end of, and following treatment completion. Although there was a small drop-off in spiritual beliefs at the 3-month follow-up, overall, spiritual beliefs were at a higher level than prior to treatment. These findings indicate that the inclusion of the spiritual component of the Bridge Programme may be effective in producing change in terms of increasing spiritual beliefs. Furthermore, similar to previous research (Leigh et al., 2005) an increase in spiritual beliefs was associated with a decrease in severity of alcohol or drug use. An association between increased spirituality and decreased severity of use does not necessarily imply causality (e.g., increased spirituality driving reduction in substance use) or direction of causality. For example, it may be that with the reduction of severity of substance use there is space for spirituality to unfold or grow (Segal, 2020).

Similarly, the qualitative data also demonstrated that for many participants, the treatment programme was seen as increasing spiritual wellbeing and that spirituality had a range of definitions. For a small group of participants, however, developing spirituality was not considered a part of the recovery process and in some cases was seen as an unwanted element of the programme. An important consideration that may have framed participants’ answers to the open-ended questions is how they understood the concept of spirituality.

That participants in the present study defined spirituality in a number of ways is in keeping with previous research findings (see Segal, 2020, for overview). These findings fit with other New Zealand health research (Egan et al., 2011) and international health research (de Brito Sento et al., 2021; Puchalski et al., 2014), demonstrating that the spiritual concept is understood as plural, broad, and for many, eclectic (de Brito Sento et al., 2021; Flannelly et al., 2002; Joint Commission on Accreditation of Healthcare Associations, 2005; Tanyi, 2002).

Spirituality is considered by many existing studies as a protective factor (Hodge, 2001; Larson & Larson, 2003; Miller, 1999; Ritt-Olson et al., 2004) and one that improves AOD outcomes (Avants et al., 2001; Hai et al., 2019). It is fair to propose that such findings are confirmed in this evaluation with both the WHOQoL-SRPB results and the qualitative findings suggesting improved outcomes over the programme period. There is some criticism of the 12-Step programme’s religious focus (Galanter, 2007). However, the literature also notes that this AA approach may be a springboard into exploration of personal spirituality (Kelly et al., 2011) that is useful in the recovery process. Segal (2020) suggests AA’s approach “is that alcoholics suffer from a ‘spiritual malady’ and that a ‘spiritual solution’ is required” (p. 2). For Segal, spirituality, as found in the Bridge Programme evaluation (Patterson et al., 2015), is understood broadly, across the religious—secular continuum.

The findings of this programme evaluation, particularly related to the positive changes regarding spirituality and the spiritual practices (such as Recovery Church, prayer, and medication), concur with the literature, affirming the Bridge Programme as an opportunity for spiritual growth that has a positive impact on recovery, at least for the evaluation period examined here.

Although the present study demonstrates spiritual growth occurs in an AOD programme and is associated with recovery, the exact mechanism as to the way in which spirituality aids AOD recovery remains unanswered. There is some speculation that addiction is a response to a personal and/or societal spiritual void (McCoy et al., 2005) or vacuum. And in fact, in the present study, one client suggested “*I have found some of my soul*” _(310)_, which implies that it was lacking. This is an area that could be further explored in subsequent evaluations by asking questions around whether there is spiritual pain, distress, or a void that is being attempted to be filled with the misuse of AOD.

Aotearoa New Zealand has recently been through a national mental health and addictions review (New Zealand Government, 2018), which calls for a more holistic approach to recovery and the inclusion of spirituality in the process. Similarly, Māori and Pasifika approaches highlight the place of spirituality in wellbeing (Capstick et al., 2009; Valentine et al., 2017). In this context, based on the evaluation, the Bridge Programme, which is described by The Salvation Army as a “distinctly spiritual journey” and “a practical expression of its [The Salvation Army’s] Christian-based love and concern for all people,” lives up to its aims. This suggests that more focus on generic spiritual approaches are warranted, which might include spiritual care related to nature, culture/wairuatanga, the arts, sport, family/whānau and so on, things that provide meaning and purpose, a reason for living free of the fog of AOD.

### Limitations

The findings of the present study need to be considered in light of the study’s limitations. Although we measured spirituality before and after treatment, measures of spirituality were subjective and via self-report. Further, in the qualitative component of this study, participants’ subjective definitions of spirituality were administered post-treatment only. It would have been interesting to compare pre- and post-treatment views of spirituality in terms of how the treatment programme may have shaped or altered these views. Another point to consider is that although the participants in the present study were generous and forthcoming in their written responses to open-ended questions on spirituality, face-to-face semi-structured open-ended interviews may have provided richer data. Additionally, the results of the present study only considered those who completed a “dose” of treatment and we cannot compare the impact of spirituality for those who did not complete a dose of treatment (e.g., did spirituality increase for those who received partial treatment? Was spirituality a factor in improvement or drop out for those who left the programme early?). Future research that follows up on those who leave a treatment programme before having received “a dose” of treatment would help clarify spirituality-related matters for those leaving treatment programmes early. A further limitation of the evaluation was the lack of inclusion of specifically culturally-related questions, for example, questions asking participants about tikanga Māori aspects of the programme, though as noted, there were very few qualitative results of this nature.

## Conclusions

Spirituality is a key component of The Salvation Army’s Bridge Programme Model of Treatment. Explicitly this is expressed through Recovery Church, prayer, spirituality lifters/classes, and the higher power component of the 12-Step programme. There are also subtle spiritual aspects of the programme such as focusing on meaning and purpose beyond addiction, a broader “spiritual solution” approach (Segal, 2020, p. 2). The majority of participants held a broad understanding of spirituality, with a small but committed group equating it more directly with religion. Participants who completed a therapeutic dose of the Bridge Programme had statistically significant increases in spiritual wellbeing. These increases were maintained at follow-up. Furthermore, an increase in spiritual wellbeing was associated with a decrease in severity of alcohol and drug use.

Most participants said that spirituality was important and that it had positively changed over the time of the programme. Many participants suggested that their belief system and what mattered most to them was their sobriety and abstinence. Self-awareness, including a general awareness of a generic spirituality, was an important theme in participants’ responses. Another strong theme across all questions was family/whānau, that is, the importance of family in participants’ life, how much they mattered to the participant.

The present evaluation demonstrated that the spiritual components of the Bridge Programme were widely valued and deemed helpful. Given its obvious importance, development of the explicit spiritual aspects of recovery need further research and evaluation to build this area of evidence informed addiction processes to help facilitate sobriety, self-awareness and ultimately help those of us challenged by addiction to contribute positively to society.

## Footnotes

1 Participants could identify more than one ethnic group.
